# School Fevers

**Published:** 1894-04-21

**Authors:** 


### The Hospital

An Institutional Journal of

The Medical Sciences and Hospital Administration, with

AN EXTRA NURSING SUPPLEMENT.

Vol. XVI.—No. 395.] [April 21, 1894.

### School Fevers.

That clinical knowledge is still our most trustworthy knowledge, and that clinical experience is our safest guide in practice, is convincingly demonstrated in a small but carefully written monograph just given to the profession by Dr. Clement Dukes, Medical Officer to Rugby School. Dr. Dukes undertakes to convince us that epidemic roseola, or German measles, is a quite distinct disease from morbilli, or English measles, and quite distinct also from scarlet fever. The chief reasons why he is so anxious to convince us are two: in the first place, he desires to save boys from unnecessary imprisonments and dangers, and their parents from alarm; and, in the second, he is concerned to preserve the honour of medicine as an accurately scientific profession.

Now, if the science of bacteriology were all that it sometimes thinks it is, and all that we hope it will one day become, we need not be dependent upon the very roundabout method of clinical observation, described by Dr. Dukes, for accurate knowledge. All that we should require in order to enable us to decide at once whether a given case was roseola, morbilli, or scarlatina would be to examine the micro-organisms present in the blood or elsewhere, and perhaps by way of certain confirmation to carry out a culture, or an inoculation on a suitable animal. We should then feel as sure of our ground as the mathematician who has just brought the solution of his problem to demonstrable completion. This is what bacteriology does not as yet enable us to do, though it certainly should, and we hope it some day will. It is a little startling, however, in these days of microbiology to be thrown back so entirely upon the old-fashioned methods of the bedside. Dr. Dukes' monograph reads like a score of pages taken out of a very carefully prepared and intelligent text-book of twenty years ago. But knowledge is knowledge, however it be obtained; and careful observations and comparisons made at the bedside, in the absence of laboratory aids, satisfy at least some of the legitimate canons of true Baconian observation and experimentation.

The real things which the working practitioner and the scientific observer want to know are these: Whether there be a true and actual distinction between common measles and roserash on the one hand, and between roserash and scarlet fever on the other. If this is established, then we need assurance as to the essential points of difference, in order that we may be able always to say with precision whether a given case is one of roserash, common measles, or scarlet fever. In the third place we wish to know with equal certainty what is the complete history of each disease in a normal type, in order that we may understand not only how to manage any case for its own safety and restoration to health, but also how we may do this at the least inconvenience to the patient and others. And, finally, the all-important question of isolation and the spread of infection is clearly dependent upon exact diagnosis and a knowledge of the varying course and possibilities of each of the three diseases.

Does Dr. Dukes succeed in establishing to demonstration the fact that there is a disease, roserash, which in its origin, essential characteristics, history, and possibilities differs *toto coelo* on the one hand from common measles, and on the other from scarlet fever? He thinks he does; and we are quite certain that many medical men reading his monograph will think so with him. For our own part we feel that, as in the case of the differentiation between croup and diphtheria, the possibilities of error are so manifold that we confess ourselves to be still in a state of doubt. Can the question be really decided with certainty at the bedside? Is not this just one of those classes of instances which demand for scientific certainty the intervention of the physiologico-pathological laboratory? We have affirmed that the most trustworthy knowledge we now possess is clinical knowledge; but that is a very different thing from stating that the clinical knowledge we possess is everything we could desire. On the contrary—and nothing better brings out the fact than an able and thoroughly scientific clinical monograph like that of Dr. Dukes—the want of microbial demonstration and of inoculatory confirmation leaves us still in doubt; in doubt in a class of cases where certainty would not only conserve happiness and diminish cost, but would save innumerable lives.

In the meantime, however, the laboratory gives us no manner of help in the very large and important class of fevers under discussion. We are, therefore, perforce, thrown back upon clinical methods of diagnosis and treatment. Our choice is not between clinical knowledge and laboratory knowledge, but between clinical knowledge and no knowledge at all. So far as clinical observation and experience can go Dr. Dukes seems to have gone. His opportunities for twenty-five years have been full and varied. If a medical man who has been in professional charge of one of our largest public schools for nearly a quarter of a century has nothing of importance to tell us about the fevers of boyhood he must be either an exceedingly dullwitted person or the methods of clinical research must be of extremely little scientific worth. As a matter of fact Dr. Dukes tells us that it is his experience of twenty-five years, and the errors he has fallen into and renounced, with the errors he has seen other practitioners fall into, without renouncing, which have driven him to the making of all manner of careful observations for the purpose of amassing definite knowledge, and to the resolution to bring his definite conclusions before the members of the profession, in order that they may cease from their own professional errors, and put an end to the manifold practical inconveniences which spring therefrom.

Morbilli, or roserash, Dr. Dukes assures us, is neither so dangerous nor so prolonged a disease as either common measles or scarlet fever. But it has this unlucky peculiarity, that it puts on the outward appearance of both these diseases. The eruption of roserash, even to the experienced observer, sometimes looks like that of measles and sometimes like that of scarlet fever. So much is this the case that many a boy suffering from roserash has been treated as if suffering from scarlet fever, has endured all the worries and inconveniences of such unnecessary treatment, and besides has had to undergo six or eight weeks of imprisonment in an isolation ward when three weeks, or even less, would have been ample. Now consider such facts as these in their application to a public school of five or six hundred boys, in their application still more to hundreds of public and private schools, in their application most of all to all the schools of every class and to all the boys and girls in the land. What infinite possibilities are here opened out of putting boys with roserash into scarlet fever wards, and so of spreading scarlet fever in hundreds of directions where otherwise it would not be spread! What possibilities also of closing schools for the greater part or even the whole of a term for scarlet fever when, if the more innocent fever were accurately diagnosed, the school need not be closed at all!

The more purely technical aspects of the case we have left ourselves no space to discuss, nor is it necessary, since Dr. Dukes himself has exhausted the resources of clinical possibility. Perhaps the best result of reading such a monograph is the sense of profound dissatisfaction it excites in the scientific medical mind. If every medical man in the kingdom could be certainly convinced that roserash is a disease *sui generis*, and quite distinct always and everywhere from either scarlet fever or measles, and if, in addition, he had a simple but absolutely trustworthy method of differentiating among the three, a change would be wrought in school life and school experience which can only be properly described as a revolution. The subject is one of instant practical importance. Dr. Dukes has given the clinician an imperative call to new studies and new observations. To the laboratory worker his monograph should be uncomfortable reading. If so much has been done in the regions of typhoid, anthrax, and cholera, why has little or nothing of a practical kind been done in the department of those much more urgent fevers and diseases which are so common that they come and go with the seasons?

